# Machine learning developed an immune evasion signature for predicting prognosis and immunotherapy benefits in lung adenocarcinoma

**DOI:** 10.3389/fcell.2025.1622345

**Published:** 2025-06-19

**Authors:** Dongxiao Ding, Gang Huang, Liangbin Wang, Ke Shi, Junjie Ying, Wenjun Shang, Li Wang, Chong Zhang, Maofen Jiang, Yaxing Shen

**Affiliations:** ^1^ Department of Thoracic Surgery, The People’s Hospital of Beilun District, Ningbo, Zhejiang, China; ^2^ Department of Anorectal Surgery, Health Science Center, The People’s Hospital of Beilun District, Ningbo University, Ningbo, Zhejiang, China; ^3^ Department of Thoracic Surgery, First Affiliated Hospital, School of Medicine, Zhejiang University, Hangzhou, Zhejiang, China; ^4^ Department of Pathology, The People’s Hospital of Beilun District, Ningbo, Zhejiang, China; ^5^ Department of Thoracic Surgery, Zhongshan Hospital, Fudan University, Shanghai, China

**Keywords:** immune escape, machine learning, lung adenocarcinoma, prognostic signature, immunotherapy

## Abstract

**Background:**

Lung adenocarcinoma (LUAD) is one of the most common cancers worldwide and a major cause of cancer-related deaths. The advancement of immunotherapy has expanded the treatment options for LUAD. However, the clinical outcomes of LUAD patients have not been as anticipated, potentially due to immune escape mechanisms.

**Methods:**

An integrative machine learning approach, comprising ten methods, was applied to construct an immune escape-related signature (IRS) using the TCGA, GSE72094, GSE68571, GSE68467, GSE50081, GSE42127, GSE37745, GSE31210 and GSE30129 datasets. The relationship between IRS and the tumor immune microenvironment was analyzed through multiple techniques. *In vivo* experiments were performed to investigate the biological roles of the key gene.

**Results:**

The model developed by Lasso was regarded as the optional IRS, which served as an independent risk factor and had a good performance in predicting the clinical outcome of LUAD patients. Low IRS-based risk score indicated higher level of NK cells, CD8^+^ T cells, and immune activation-related functions. The C-index of IRS was higher than that of many developed signatures for LUAD and clinical stage. Low risk score indicated had a lower tumor escape score, lower TIDE score, higher TMB score and higher CTLA4&PD1 immunophenoscore, suggesting a better immunotherapy response. Knockdown of PVRL1 suppressed tumor cell proliferation and colony formation by regulating PD-L1 expression.

**Conclusion:**

Our study developed a novel IRS for LUAD patients, which served as an indicator for predicting the prognosis and immunotherapy response.

## 1 Introduction

Lung cancer is one of the most common cancers in the United States and a major cause of cancer-related deaths, with an estimated 234,580 new cases and 125,070 deaths each year (Siegel et al., 2024). Lung adenocarcinoma (LUAD) is the most common subtype of lung cancer, accounting for approximately 40% of all lung cancer cases (Song et al., 2021; Xu and Lu, 2024). For early-stage LUAD cases, surgical resection remains the primary treatment method However, there is usually have no obvious clinical symptoms in the early stages of LUAD cases, which leads to a significant proportion of LUAD cases being diagnosed at an advanced stage, thus missing the best opportunity for surgical intervention. Although there are multiple treatment options available for advanced lung cancer cases, including chemotherapy and targeted therapy, their clinical outcomes remain unsatisfactory, with a 5-year overall survival rate of only 4% for patients with metastatic lung cancer (Lahiri et al., 2023). However, in recent years, immunotherapy has provided more possibilities for extending the lives of patients with inoperable lung cancer. Nowadays, several immunotherapy drugs have been approved for first-line treatment of non-small cell lung cancer, including nivolumab and pembrolizumab (Provencio et al., 2023; Gandhi et al., 2018). However, a significant number of patients do not respond to immunotherapy throughout the treatment period. Currently, reliable biomarkers for predicting prognosis and immunotherapy sensitivity are limited, and further research is needed.

The efficacy of immunotherapy for LUAD remains limited, mainly due to the tumor heterogeneity and immunosuppressive microenvironment within the tumor (Passaro et al., 2022; Li et al., 2024). The lungs have an immunosuppressive tendency that can weaken T-cell-mediated antigen responses (Tanoue et al., 2023). To maintain normal physiological homeostasis, the immunosuppressive effect of the lungs is maintained by other resident immune cells, including dendritic cells (DCs) and regulatory T cells (Tregs) (Mengistu et al., 2024). However, in advanced lung adenocarcinoma, this immunosuppressive environment facilitates tumor immune escape, allowing cancer cells to evade host immune clearance. Intrinsic tumor immune escape refers to the strategies used by tumor cells to avoid host immune detection and destruction, thereby promoting their survival, proliferation, and resistance to immunotherapy. In advanced tumor patients, monocytes highly express CD48, a molecule that acts on natural killer (NK) cells, causing them to become rapidly and transiently activated before becoming exhausted and eventually dying (Wu et al., 2013). Tumor-associated macrophages are key regulatory cells in the immune response of lung adenocarcinoma, promoting tumor immune escape by secreting inhibitory programmed death ligand 1 (PD-L1) to suppress T-cell activation and function (Singhal et al., 2019). Therefore, exploring immune escape-related genes in the immune microenvironment of LUAD and establishing relevant models to predict the efficacy of immunotherapy is of great significance for the development of immunotherapy.

In the current study, machine learning was performed to construct and verify an immune evasion related genes-based signature (IRS) for LUAD using TCGA, GSE72094, GSE68571, GSE68467, GSE50081, GSE42127, GSE37745, GSE31210 and GSE30129. The role of IRS in predicting the immunotherapy response of cancer patients were also explored.

## 2 Materials and methods

### 2.1 Datasets and gene sets

We obtained bulk RNA-seq data from the TCGA database for LUAD cases (n = 503) and normal lung cases (n = 59). The predictive value of IRS was further assessed using eight GEO datasets: GSE72094 (n = 398), GSE68571 (n = 86), GSE68465 (n = 442), GSE50081 (n = 127), GSE42127 (n = 173), GSE37745 (n = 106), GSE31210 (n = 226), and GSE30129 (n = 86). Additionally, two immunotherapy datasets—IMvigor210 (n = 298) and GSE91061 (n = 98)—were utilized to evaluate the ability of IRS to predict immunotherapy benefits. The immune evasion related genes (IRGs) identified in prior studies are presented in Supplementary Table S1 (Lawson et al., 2020; Wen et al., 2025). The detail for the data preprocessing and normalization procedures were provided in the Supplementary Material.

### 2.2 Integrative machine learning based IRS

The “limma” package was employed to detect differentially expressed genes (DEGs) in LUAD among IRGs, using |LogFC| ≥ 1.5 as the threshold. Potential prognostic biomarkers were identified through univariate Cox regression analysis. To establish a robust prognostic IRS, these biomarkers were subjected to an integrative machine learning analysis, which included methods such as Lasso, RSF, Ridge, CoxBoost, stepwise Cox, Enet, plsRcox, GBM, SuperPC and survival-SVM. Within the leave-one-out cross-validation framework applied to the TCGA dataset, the candidate genes for the prognostic model and their corresponding coefficients were determined. Subsequently, Harrell’s concordance index (C-index) for all prognostic models was calculated using the TCGA dataset and eight GEO datasets. Similar machine learning methodologies have been described in prior studies (Liu et al., 2022a; Liu et al., 2022b; Zhang et al., 2022). Detailed parameter tuning information for the R scripts used in this study can be found on the Github repository (https://github.com/Zaoqu-Liu/IRLS). The detailed parameter of each machine learning method was shown in Supplementary Material. The prognostic IRS with the highest average C-index was selected as the optimal model. Using the “surv_cutpoint” function from the R package “survminer,” the optimal cutoff value was identified, allowing us to classify LUAD cases into low and high risk score groups across all datasets. Univariate and multivariate Cox regression analyses were conducted to identify risk factors associated with LUAD prognosis. The C-index curves of clinical characteristics and 100 previously developed prognostic signatures for LUAD (Supplementary Table S2) were computed using the “rms” package. Finally, a predictive nomogram was constructed using the R package “nomogramEx,” integrating both the IRS and clinical characteristics.

### 2.3 Immunotherapy analyses and gene set enrichment analyses

Seven algorithms, including CIBERSORT, MCPcounter, QUANTISEQ, XCELL, CIBERSORT-ABS, TIMER, and EPIC, were used to investigate the correlation between IRS and immune cells with R package “immunedeconv” (Li et al., 2020). The immune and ESTIMATE scores for tumor cases were computed using the R package “estimate” (Yoshihara et al., 2013). After obtaining the cancer-related hallmark gene set from the Molecular Signatures Database, we performed single-sample Gene Set Enrichment Analysis (ssGSEA), which was also used to assess the scores of immune cells, immune-related functions, and tumor escape and surveillance in LUAD patients. The IC50 values of drugs for LUAD patients were calculated using the R package “oncoPredict” and data from the Genomics of Drug Sensitivity in Cancer database (https://www.cancerrxgene.org/). Several tools were employed to evaluate the role of IRS in predicting immunotherapy benefits. The Tumor Immune Dysfunction and Exclusion (TIDE) and tumor mutation burden (TMB) score for LUAD patients were retrieved from the TIDE platform (http://tide.dfci.harvard.edu). The Immunophenoscore (IPS) for LUAD patients was downloaded from the Cancer Immunome Atlas (TCIA, https://tcia.at/home).

### 2.4 Cell lines and knockdown of PVRL1

Normal lung cell line (HBE) and LUAD cell lines were obtained from Shanghai Institute of Biochemistry and Cell Biology (Shanghai, China). ATCC recommended medium and fetal bovine serum (FBS; Gibco) and 1% penicillin-streptomycin (Sigma-Aldrich, St. Louis, United States) were used to calculated these cells, which were maintained in circumstances containing 5% CO2 and 95% saturated humidity at 37°C. Lipofectamine 3000 transfection reagent (Invitrogen) was used for the transfection of A549 and H1299 with NCAPG shRNA lentivirus (PVRL1-sh) and shRNA lentivirus vector (sh-NC) from GeneChem (Shanghai, China) based on the manufacturer’s instructions.

### 2.5 RT-qPCR and Western blotting

RNA was extracted from cells using TRIzol (Takara Bio) and subsequently reverse-transcribed into cDNA with an oligo (dT) primer. RT-qPCR was performed using SYBR Premix Ex Taq (Takara Bio) on the ABI 7900HT detection system (Thermo Fisher Scientific Inc.), with gene expression levels normalized to the internal control GAPDH. Proteins were extracted from cell lines using lysis buffer (Beyotime), and their concentrations were determined using a bicinchoninic acid (BCA) kit (Beyotime). The proteins were denatured by boiling in loading buffer (Beyotime) for 3 min before being separated via SDS-PAGE and transferred onto a PVDF membrane (Thermo Fisher Scientific, United States). The membrane was blocked with 5% BSA to minimize non-specific binding. Primary antibodies were incubated overnight at 4°C, followed by secondary antibodies for 1 h at room temperature. Protein bands were visualized using enhanced chemiluminescence (ECL) and the ABC system, and their intensities were quantified with ImageJ software (National Institutes of Health, United States), using GAPDH as the loading control.

### 2.6 Proliferation, colony formation, and wound scratch assay

To assess cell proliferation, LUAD cell lines were seeded in 96-well plates at a density of 3,000 cells per well (in triplicate). Cell Counting Kit-8 (Beyotime) was added to the wells at 4, 24, 48, 72, and 96 h. The proliferation index was determined by calculating the ratio of the OD value at each specified time point followed the method in the previous study (Yang et al., 2025; Qiu et al., 2024), LUAD cells were plated in 6-well plates at a density of 500 cells per well in colony formation assay. The following day, the cells were treated with bortezomib in culture medium for 2 weeks. Afterward, the cells were washed twice with PBS, fixed with 4% paraformaldehyde for 30 min, and stained with 0.1% crystal violet for 15 min. Following this, the cells were rinsed twice with PBS and imaged using a digital camera.

The wound scratch assay was conducted to evaluate the migratory ability of LUAD cells. Images of the scratched monolayer were captured at 0 h and 48 h using a digital camera system (Olympus Corporation, Tokyo, Japan).

### 2.7 Statistical analysis

Correlation analysis between two continuous variables was performed using Pearson’s rank correlation methods. Differences between continuous variables were assessed using either the Wilcoxon rank-sum test or the Student’s t-test, as appropriate. The two-sided log-rank test was applied to evaluate differences in Kaplan-Meier survival curves across groups. All statistical analyses were conducted using R software (version 4.2.1).

## 3 Results

### 3.1 Identification of potential prognostic biomarkers for LUAD patients

Compared with normal tissues, a total of 7,906 genes were differently expressed in LUAD tissues (Supplementary Figure S1A). Supplementary Figure S1B showed the overlap between IEGs and DEGs, identifying 60 genes were differently expressed IEGs. As shown in Supplementary Figure S1C, additional univariate Cox analysis revealed that 10 genes were significantly linked to the overall survival rate of LUAD (p < 0.05). These genes included TRAF2, STAT1, SMG7, PVRL1, NPLOC4, HDAC1, FADD, CEP55, BOLA3, and AHSA1.

### 3.2 Integrative machine learning algorithms developed a stable IRS

**FIGURE 1:**
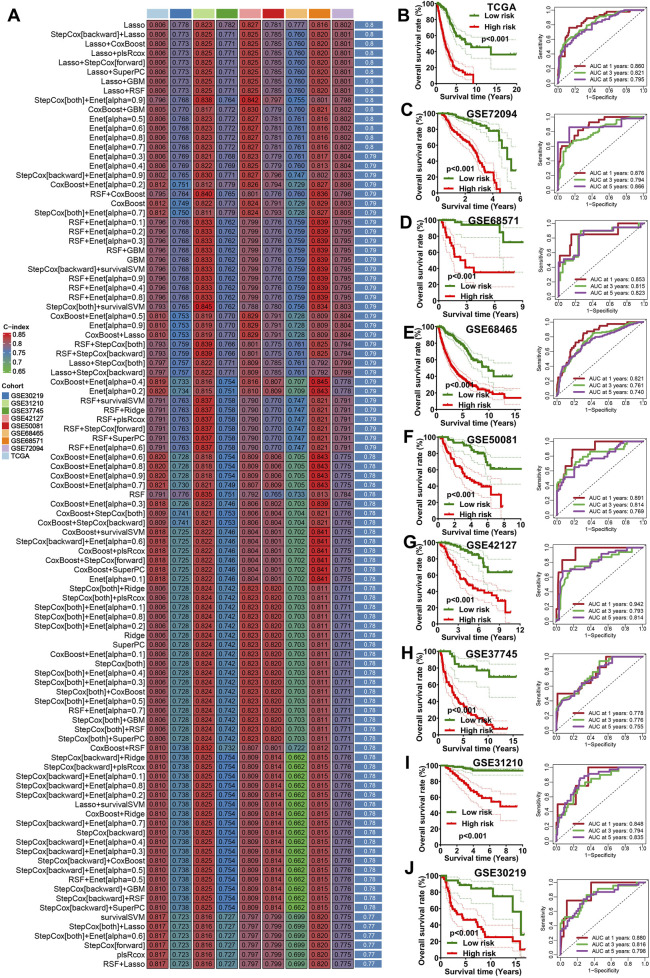
Machine learning developed an immune escape-related signature. **(A)** The C-index of 101 kinds prognostic models in all the datasets. The survival curve of different risk score groups and their corresponding ROC curve in TCGA cohort **(B)** and 8 GEO cohort **(C–J)**.

The 10 potential prognostic biomarkers were subjected to an integrative machine learning process involving the 10 methods mentioned earlier to construct a stable IRS. As a result, a total of 101 prognostic models were generated and the average C-index values of each model was shown in Figure 1A. The prognostic model developed using Lasso exhibited the highest average C-index of 0.8 and was thus selected as the optimal prognostic IRS. This optimal IRS was established based on 5 IEGs, and the risk score for LUAD cases were calculated using the formula: risk score = 0.0010×CEP55^exp^ + 0.0050×TFAF2^exp^ + 0.1231×PVRL1^exp^ + 0.0476×FADD^exp^ + 0.0053×AHSA1^exp^ + 0.0003×STAT1^exp^. LUAD patients were categorized into high and low IRS score groups using the optimal cutoff value. As shown in Figures 1B–J, LUAD patients with high risk score demonstrated poorer overall survival (OS) rates across multiple datasets, including TCGA, GSE72094, GSE68571, GSE68467, GSE50081, GSE42127, GSE37745, GSE31210 and GSE30129 (all p < 0.05). The corresponding 1-, 3-, and 5-year AUC values were as follows: 0.860, 0.821, and 0.795 in the TCGA cohort; 0.876, 0.794, and 0.866 in the GSE72094 cohort; 0.853, 0.815, and 0.823 in the GSE68571 cohort; 0.821, 0.761, and 0.740 in the GSE68465 cohort; 0.891, 0.814, and 0.769 in the GSE50081 cohort; 0.942, 0.793, and 0.814 in the GSE42127 cohort; 0.778, 0.776, and 0.755 in the GSE37745 cohort; 0.848, 0.794, and 0.835 in the GSE31210 cohort; and 0.880, 0.816, and 0.798 in the GSE30219 cohort (Figures 1B–J).

### 3.3 IRS showed good performance in predicting the prognosis of LUAD patients

**FIGURE 2:**
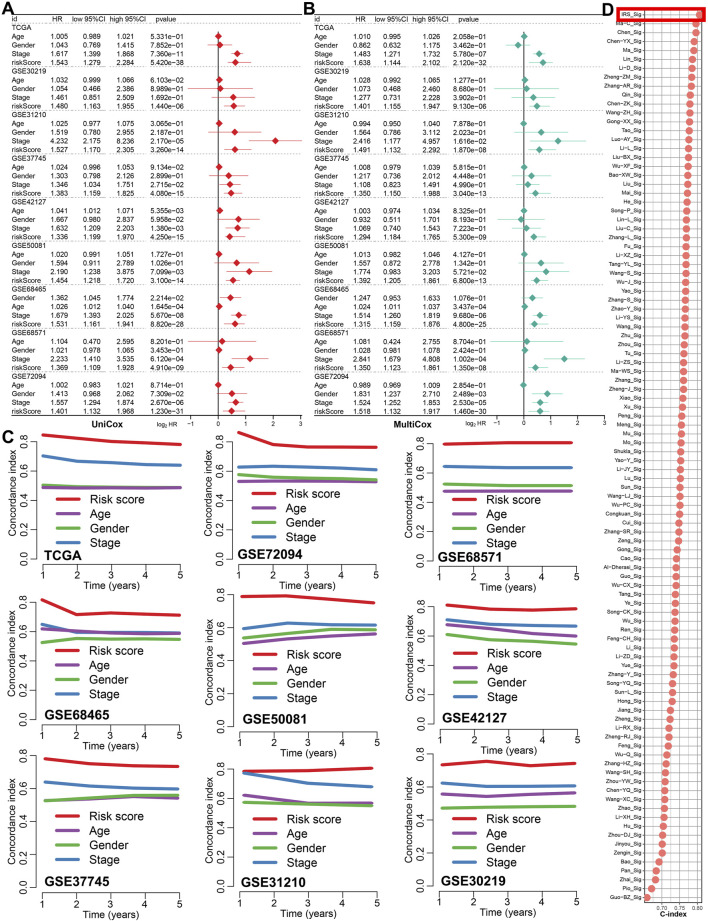
The predicting performance of IRS in the prognosis of LUAD patients. **(A,B)** Univariate and multivariate Cox regression analyses identified IRS-based risk score as a risk factor for the prognosis of LUAD patients. **(C)** The C-index was used to compare the predictive value of IRS, age, gender, and clinical stage for LUAD prognosis in the TCGA and 8 GEO cohort. **(D)** The C-index was also utilized to compare the prognostic performance of IRS with 100 other established signatures for LUAD patients.

**FIGURE 3:**
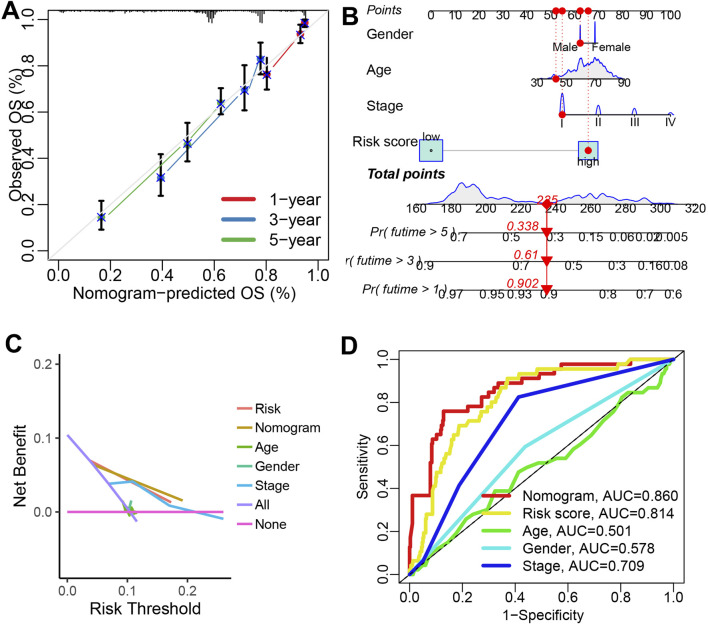
Construction of a predictive nomogram. **(A)** A nomogram was established using IRS, age, gender, and clinical stage as variables. **(B,C)** Calibration plots and ROC curves were used to assess the performance of the nomogram in predicting the clinical outcomes of LUAD patients. **(D)** A DCA curve illustrated the strong clinical applicability potential of the nomogram.

Figures 2A,B showed the result of univariate and multivariate cox regression analysis in all the GEO and TCGA cohorts, indicating IRS as an independent risk factor for the overall survival of LUAD patients (all p < 0.05). Compared to age, gender, and clinical stage, the C-index of the IRS was found to be higher across all GEO and TCGA cohort (Figure 2C). Numerous prognostic signatures have been developed for LUAD. To evaluate the predictive performance of our IRS relative to other signatures, we gathered 100 prognostic signatures randomly and computed their respective C-index (Supplementary Table S2). As illustrated in Figure 2D, the C-index of our IRS was superior to these signatures within the TCGA cohort. For predicting the clinical outcomes of LUAD, we subsequently constructed a nomogram incorporating clinical stage, IRS, gender, and age (Figure 3A). There was a strong agreement between the predicted curve and the ideal curve (Figure 3B). The AUC of the nomogram was greater than that of IRS, age, gender, and clinical stage individually (Figure 3C). Additionally, the DCA curve indicated that the nomogram provided better predictive utility compared to risk score, tumor grade, and clinical stage (Figure 3D).

### 3.4 The tumor microenvironment difference in patients with different risk score

**FIGURE 4:**
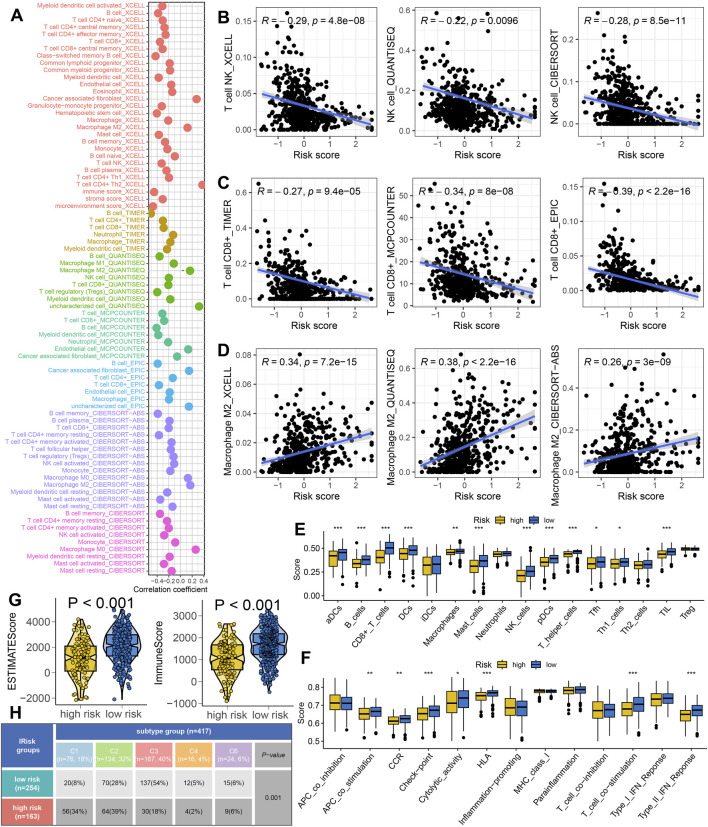
The tumor microenvironment landscape in different risk score group. **(A)** Seven algorithms-based correlation between risk score and the abundance of immune cell. **(B–D)** High risk score indicated higher the abundance of NK cells, CD8^+^ T cells and lower level of macrophage M2. **(E,F)** The level of immune cells and immune-related functions in different risk score groups. **(G)** The immune score and ESTIMAE score in different risk score groups. **(H)** The immune landscape in different IRS score groups. *p < 0.05, **p < 0.01, ***p < 0.001.

Figure 4A showed the correlation between risk score and the abundance of immune cells (p < 0.05). As shown in Figures 4B–D, the findings indicated that the IRS-based risk score was negatively correlated with the levels of NK cells and CD8^+^ T cell, and positively correlated with immunosuppressive cells macrophage M2 (all p < 0.05). Based on the data of ssGSEA analysis, there is a higher presence of mast cells, CD8^+^ T cells, B cells, DCs, and TILs in LUAD patients with low risk score (Figure 4E, all p < 0.05). Additionally, patients with low risk score demonstrated elevated immune checkpoint score, APC-co-stimulation score, T cell co-stimulation scores and cytolytic activity scores (Figure 4F, all p < 0.05). We also observed that low score group had significantly higher immune score and ESTIMATE score (Figure 4G, p < 0.001). The tumor immune landscape can be categorized into six subtypes: wound healing (C1), IFN-g dominant (C2), inflammatory (C3), lymphocyte-depleted (C4), immunologically quiet (C5), and TGF-b dominant (C6) (Thorsson et al., 2018). As shown in Figure 4H, Our data showed that low risk score group were predominantly associated with the C3 subtype, while high IRS score group were more frequently linked to the C1 and C2 subtype (p = 0.001).

### 3.5 IRS acted as an indicator for predicting immunotherapy response

**FIGURE 5:**
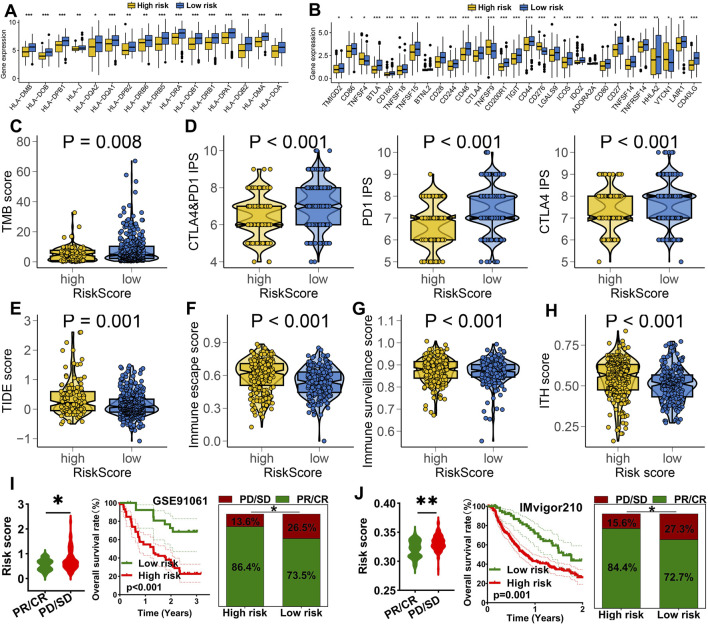
IRS acted as an indicator for immunotherapy response in LUAD. The level of HLA-related genes **(A)** and immune checkpoints **(B)** in different risk score group. Comparison of TMB score **(C)**, CTLA4&PD1 immunophenoscore **(D)**, TIDE score **(E)**, immune escape score **(F)**, immune surveillance score **(G)** and ITH score **(H)** across different risk score groups. **(I,J)** Analysis of the overall response rate and immunotherapy response rate in different risk score groups within the GSE91061 and IMvigor210 cohorts. *p < 0.05, **p < 0.01, ***p < 0.001.

Higher expression of HLA-related genes suggests a broader range of antigen presentation, which increases the probability of presenting more immunogenic antigens and enhances the likelihood of benefiting from immunotherapy (Lin and Yan, 2021). LUAD patients with low risk score exhibited higher expression levels of HLA-related genes and immune checkpoint molecules (Figures 5A,B, p < 0.05). A high TMB and IPS score is associated with a better response to immunotherapy (Liu et al., 2019; Charoentong et al., 2017). A lower TIDE score implies reduced immune escape and a better response to immunotherapy (Fu J. et al., 2020). In our study, low risk score group showed higher TMB score (Figure 5C) and elevated PD1&CTLA4 immunophenoscore (Figure 5D). They also exhibited lower TIDE scores, reduced immune escape scores, decreased immune surveillance score and intra-tumor heterogeneity score (Figures 5E–H) (all p < 0.05). These findings suggest that LUAD patients with low risk score may derive greater benefits from immunotherapy. We further evaluated the predictive value of IRS in immunotherapy using two immunotherapy-related datasets. As show in Figure 5I, the risk score was significantly higher in non-responders compared to responders in the GSE91061 cohort (p < 0.05). Moreover, a high risk score indicated a poorer overall survival rates and lower response rate (Figure 5I). Similar results were observed in the IMvigor210 cohort (Figure 5J).

**FIGURE 6:**
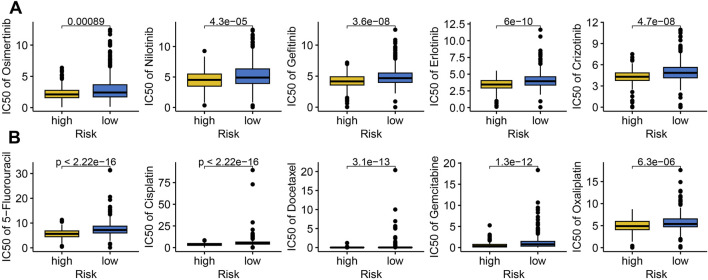
The IC50 value of drugs in different risk score group. Comparison of the IC50 value of targeted therapy **(A)** and immunotherapy **(B)** drugs across different risk score group.

Targeted therapy and chemotherapy remain critical treatments for LUAD. To assess the performance of IRS in predicting drug sensitivity in LUAD, we analyzed the IC50 values of several chemotherapeutic agents and targeted therapies. The data revealed that high risk score group had lower IC50 values for Osimertinib, Nilotinib, Gefitinib, Erlotinib, Crizotinib, 5-Fluorouracil, Cisplatin, Docetaxel, Gemcitabine, and Oxaliplatin (Figures 6A,B, all p < 0.05). This suggests that high risk score may exhibit greater sensitivity to chemotherapy and targeted therapy in LUAD.

### 3.6 Cancer related hallmarks difference in patients with different risk score

**FIGURE 7:**
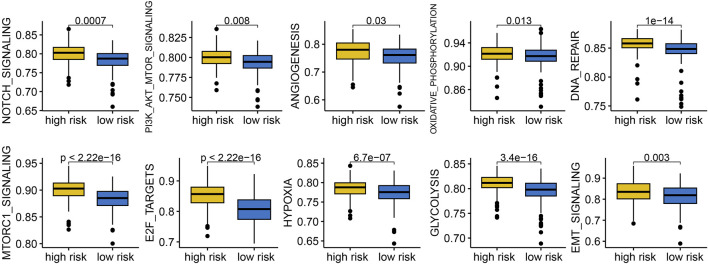
Comparison of the cancer related hallmarks difference across different risk score group.

To understand why LUAD patients with different risk score exhibited significant differences in clinical outcomes, we conducted a functional enrichment analysis. The results indicated that low risk score group indicated reduced activity of gene sets related to NOTCH signaling, PI3K-AKT-mTOR signaling, angiogenesis, oxidative phosphorylation, DNA repair, mTORC1 signaling, E2F target, hypoxia, glycolysis, and EMT signaling (Figure 7, all p < 0.05).

### 3.7 Biological functions of the selected gene

**FIGURE 8:**
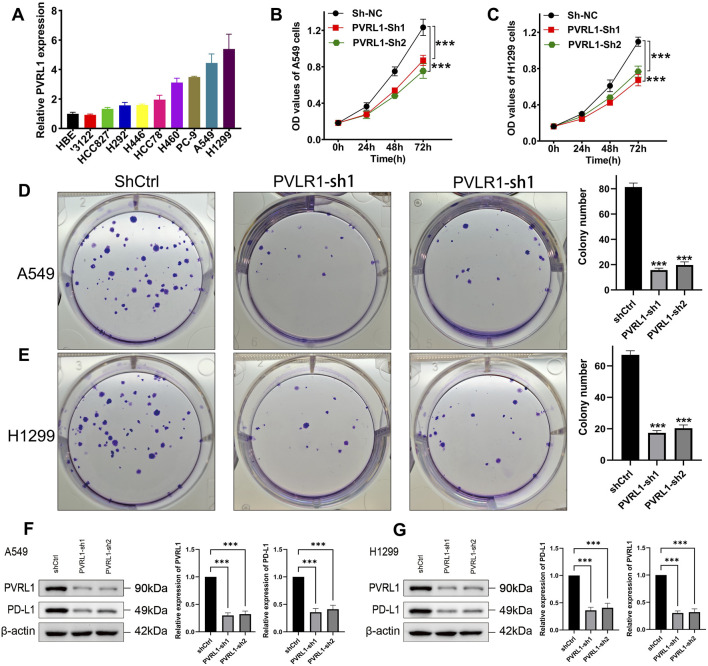
Validation of the potential function of PVRL1 in LUAD by *in vitro* assays. **(A)** Comparison of PVRL1 expressions in normal and LUAD cell lines. Knockdown of PVRL1 obviously inhibited the proliferation **(B,C)** and colony formation **(D,E)** of A549 and H1299 cells. **(F,G)** Knockdown of PVRL1 obviously inhibited the PD-L1 expression. ***p < 0.001.

To further validate the role of IRS, we focused on PVRL1, which had the most significant contribution to IRS, for additional investigation. We subsequently examined the expression levels of PVRL1 in LUAD cell lines and found that PVRL1 was highly expressed in the majority of these cell lines (Figure 8A). In subsequent experiments, the results from the CCK-8 assay indicated that silencing PVRL1 significantly suppressed the proliferation of A549 and H1299 cells (Figures 8B,C, p < 0.05). Additionally, the reduction in PVRL1 expression markedly inhibited cell migration (Supplementary Figures S2A,B, p < 0.05) and colony formation ability in both A549 and H1299 cells (Figures 8D,E, p < 0.05). We further probed into the underlying molecular mechanisms of PVRL1 in LUAD. Upon knocking down PVRL1, a decrease in PD-L1 expression was observed (Figures 8F,G). These results suggest that PVRL1 might facilitate the progression of LUAD by modulating PD-L1 expression.

## 4 Discussion

This study established an integrated machine learning framework comprising 10 different methods to construct a stable IRS for LUAD. This IRS demonstrated robust and consistent performance in forecasting the clinical outcomes of LUAD patients and functioned as an independent risk factor. Actually, previous studies have developed many prognostic signatures for LUAD. Li et al. developed an eight-gene prognostic signature for LUAD (Li et al., 2018). Another lysosomes-related gene signature acted as a prognostic marker for LUAD (Song et al., 2023). Glycolysis-related signature was correlated with survival LUAD (Zhang et al., 2019). Mo et al. developed a hypoxia-associated prognostic signature for LUAD (Mo et al., 2020). The C-index of our IRS was higher than these prognostic signatures, suggesting a better performance of IRS in predicting prognosis. The LRS were developed with the leave-one-out cross-validation method, which has some limitations. First of all, the computing cost is very high, will further restricting its clinical application. It is highly sensitive to outliers, which may cause performance fluctuations. Moreover, due to the randomness of data partitioning, the evaluation results of this method may be affected by the way the dataset is divided, leading to unstable evaluation results.

The prognostic IRS was developed using 6 IEGs, including CEP55, TRAF2, PVRL1, FADD, AHSA1, and STAT1. These IEGs also played a vital role in the development and prognosis of cancer. CEP55 was suggested as a diagnostic and prognostic biomarker for LUAD patients (Fu L. et al., 2020). Moreover, CEP55 promoted tumorigenesis and indicated the poor prognosis in endometrial cancer (Zhang et al., 2023). Oncogenic KEAP1 mutations activate TRAF2-NFκB signaling to prevent apoptosis in lung cancer cells (Deen et al., 2024). Another study found that TRAF2 favor cancer progression by promoting M2-polarized tumor-associated macrophage infiltration in renal cell carcinoma (Xu et al., 2023). Overexpression of FADD acted as a prognostic biomarker and cell proliferation in lung cancer (Chen et al., 2021; Bhojani et al., 2005). Hepatocellular carcinoma cells increase the expression of PVRL1, which suppresses cytotoxic T-cell activity through TIGIT, thereby promoting tumor resistance to PD1 inhibitors (Chiu et al., 2020). In our study, we found that PVRL1 favor tumor progression in LUAD.

Immunotherapy provides increased opportunities for extending the lives of lung cancer patients with inoperable tumors (Ruiz-Cordero and Devine, 2020). To investigate the relationship between IRS and immunotherapy response in LUAD, we utilized several evaluation metrics. Higher Tumor Mutational Burden score and intra-tumor heterogeneity score was associated with improved responsiveness to immunotherapy (Liu et al., 2019; Wang et al., 2022). The Immunophenotype score served as a more accurate predictor for the effectiveness of anti-CTLA4 and anti-PD1 antibody treatments, where a higher IPS suggested enhanced therapeutic outcomes (Charoentong et al., 2017). Additionally, a lower TIDE score implied reduced chances of immune evasion and better efficacy of immunotherapy (Fu J. et al., 2020). Our findings revealed that LUAD patients with low risk score were linked to decreased immune escape score, reduced TIDE score, elevated TMB score, and higher PD1 and CTLA4 immunophenotype score. Consequently, IRS could potentially serve as an indicator, suggesting that LUAD patients with low risk score might derive greater benefits from immunotherapy. The results of our study showed that LUAD patients with low risk score had a higher level of CD8^+^ T cells, NK cells, DCs and mast cells, as well as lower level of macrophage M2. CD8^+^ T cells are thought to induce cancer cell death mainly via perforin and granzyme (van der Leun et al., 2020). NK cells play indispensable roles in innate immune responses against tumor progression (Tang et al., 2023). DCs are a diverse group of specialized antigen-presenting cells with key roles in the initiation and regulation of innate and adaptive immune responses (Wculek et al., 2020). While M2 macrophages could promote tumor growth and invasion and inhibit the immune responses (Li et al., 2023). Thus, due to the higher level of these immune activation and tumor-killing related immune cells in LUAD patients with low risk score, they had a favorable immunotherapy benefits and prognosis.

To clarify why the clinical outcome of LUAD patients with different risk score was significantly different, we then performed functional enrichment analysis. We found that low risk score group indicated a lower sore of gene sets correlated with Notch signaling, hypoxia and angiogenesis. Notch signaling was a determinant of response to immune checkpoint blockade in LUAD (Roper et al., 2021). Hypoxia was considered as a primary trigger of angiogenesis and tumor hypoxia was correlated with a poor clinical outcome of LUAD (Goudar and Vlahovic, 2008). Angiogenesis the significantly correlated with the progression and metastasis of lung cancer (Cantelmo et al., 2020). Thus, LUAD with high risk score may be more active in the cancer-related hallmarks, resulting a poor prognosis.

Some limitations could be found in our study. All data were obtained from public databases at RNA level, prospective studies should be conducted to further verify the accuracy of IRS. The function and mechanism of PVRL1 in LUAD was further investigated through *in vivo* experiments. It would be better to incorporate classification metrics that account for class imbalance (such as F1-macro average, MCC, or balanced accuracy) rather than relying solely on continuous scores in the evaluation of immunotherapy response.

## 5 Conclusion

Our study developed a novel IRS for LUAD patients, which served as an indicator for predicting the prognosis and immunotherapy response.

## Data Availability

The original contributions presented in the study are included in the article/Supplementary Material, further inquiries can be directed to the corresponding authors.

## Supplementary material

SUPPLEMENTARY FIGURE S1 Identification of potential biomarkers in LUAD. **(A)** Heatmap showed differentially expressed genes in LUAD. **(B)** The overlap between differentially expressed genes and immune escape-related genes. **(C)** Univariate analysis identified prognostic biomarkers in LUAD.

SUPPLEMENTARY FIGURE S2 The role of PVRL1 in the migration of LUAD cells. Knockdown of PVRL1 obviously inhibited the migration of A549 **(A)** and H1299 **(B)** cells. **p < 0.01, ***p < 0.001.
