# Supplementary figures and images for: Machine learning developed an immune evasion signature for predicting prognosis and immunotherapy benefits in lung adenocarcinoma
Source: Front Cell Dev Biol. 2025 Jun 19;13:1622345. doi: 10.3389/fcell.2025.1622345 (PMC12222150; doi:10.3389/fcell.2025.1622345)

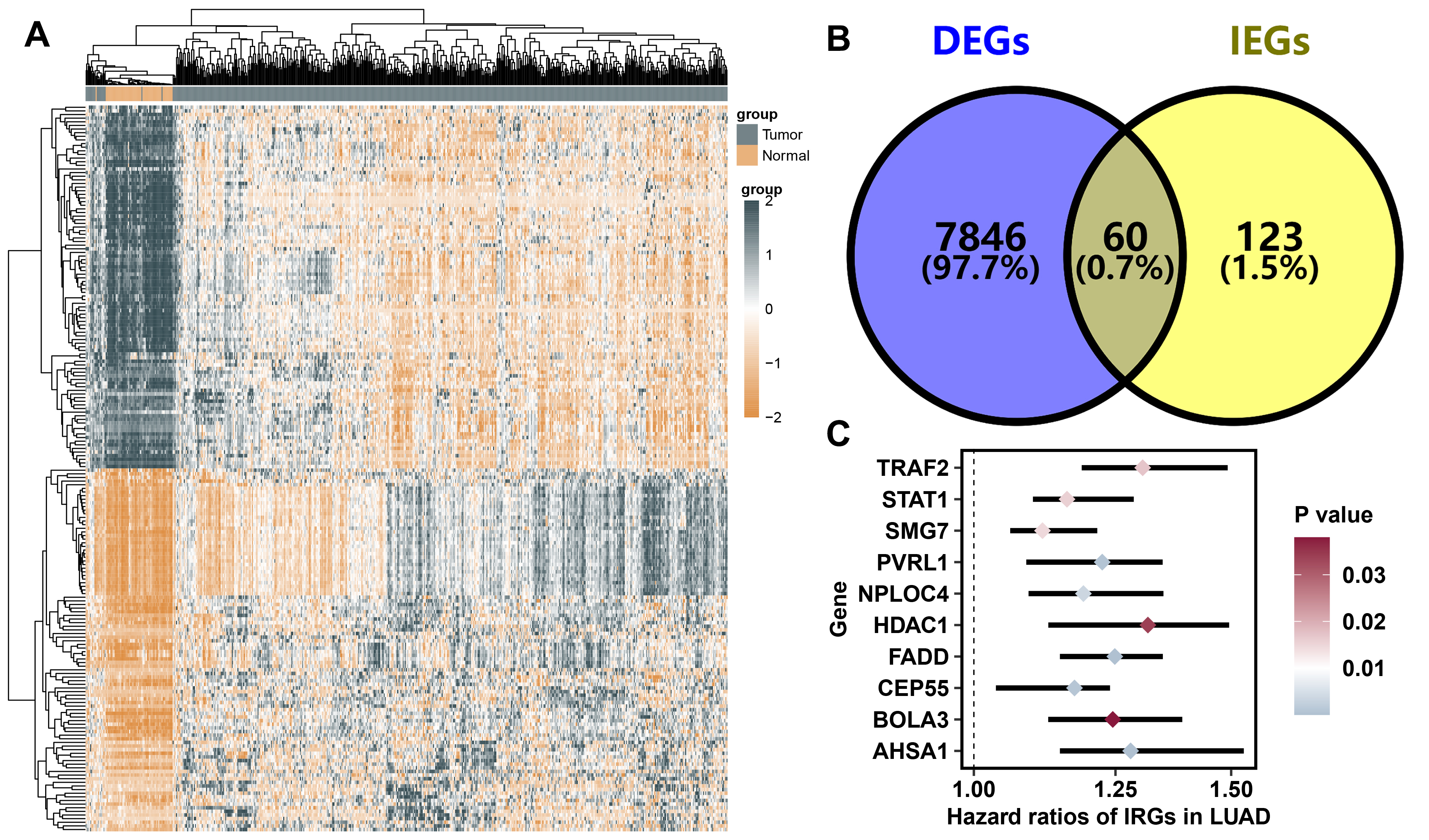

Supplement: Supplementary file 2 [file Image1.JPEG]

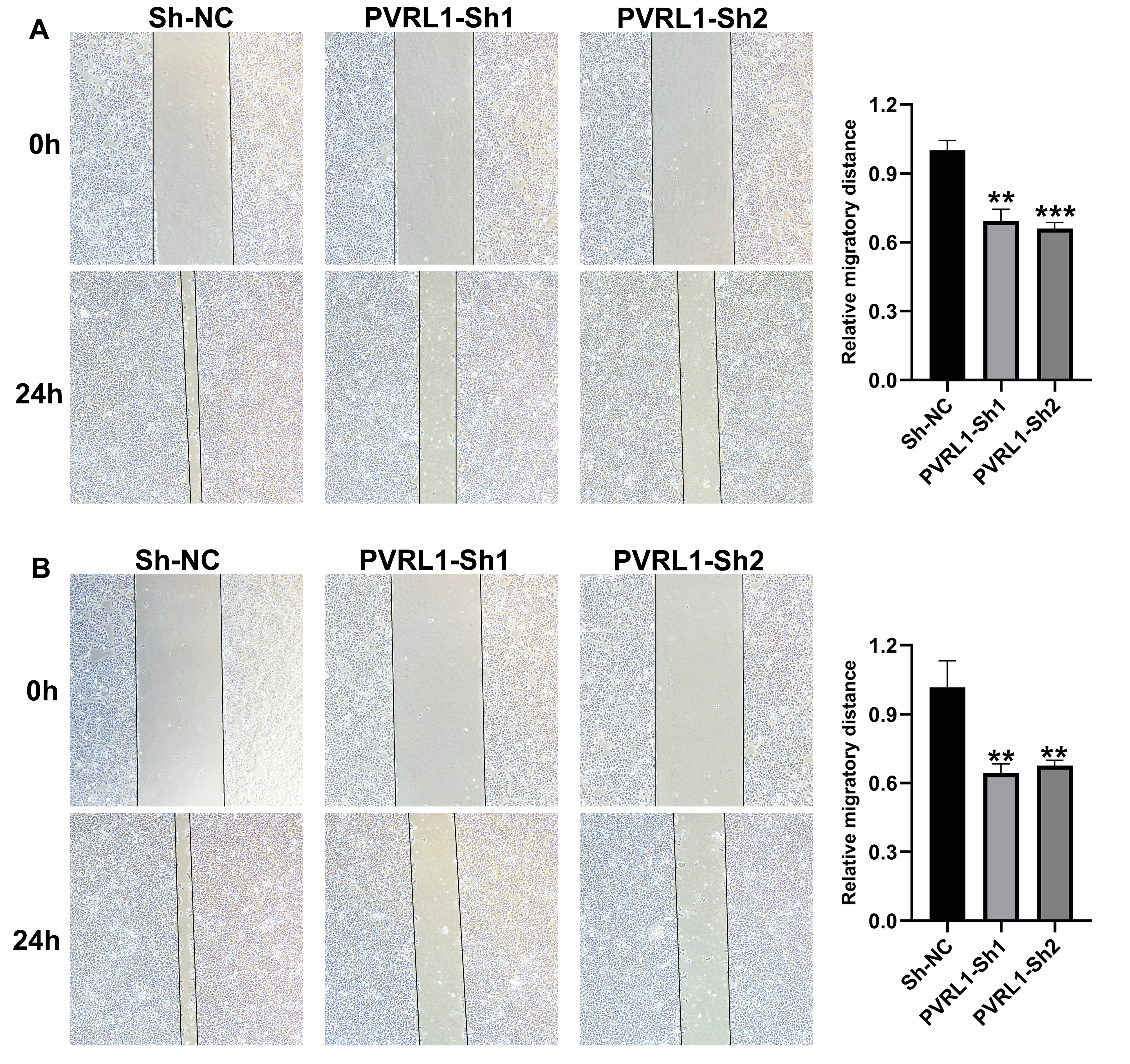

Supplement: Supplementary file 3 [file Image2.JPEG]
